# Mitotane Treatment for Malignant Leydig Cell Tumor: A Case Report

**DOI:** 10.1002/iju5.70078

**Published:** 2025-07-27

**Authors:** Daiki Katsura, Mototsugu Muramaki, Takashi Okamoto, Masaya Yamamoto, Mizuki Yutaka, Shohei Morita, Takuya Fujimoto, Yuji Yamada

**Affiliations:** ^1^ Department of Urology Kobe University Graduate School of Medicine Kobe Japan; ^2^ Department of Urology Hyogo Prefectural Amagasaki General Medical Center Amagasak Japan

**Keywords:** malignant Leydig cell tumor, mitotane, testicular neoplasms

## Abstract

**Introduction:**

Leydig cell tumors (LCTs), constituting 1%–3% of testicular tumors, are mostly benign, but malignant cases present treatment challenges. We report a malignant LCT case with a notable response to mitotane.

**Case Presentation:**

A 43‐year‐old male presented with a right testicular induration and was diagnosed with a Leydig cell tumor following orchiectomy. BEP chemotherapy was initiated for the liver metastases, but after four cycles, new lymph node and bone lesions appeared. Mitotane was started, which markedly reduced liver and nodal metastases. However, due to fatigue, anorexia, and nipple discomfort, mitotane was discontinued. The disease progressed, and the patient died 4 years post‐diagnosis and 14 months after starting mitotane.

**Conclusion:**

Mitotane shows promise in treating malignant LCTs, but careful management of adverse effects is necessary.

**Abbreviations**
HE: hematoxylin and eosin
LCT: Leydig cell tumor
RPLND: retroperitoneal lymph node dissection

**Summary**

- Mitotane has potential as a treatment for malignant Leydig cell tumors.

## 1 Introduction

Leydig cell tumors (LCTs) account for 1%–3% of all testicular tumors. Although 90% of Leydig cell tumors are benign, malignant cases can progress quickly; thus, treatment has not been adequately reported. We treated a patient with malignant LCT of the testis with mitotane, which showed a significant response.

## 2 Case Presentation

**FIGURE 1:**
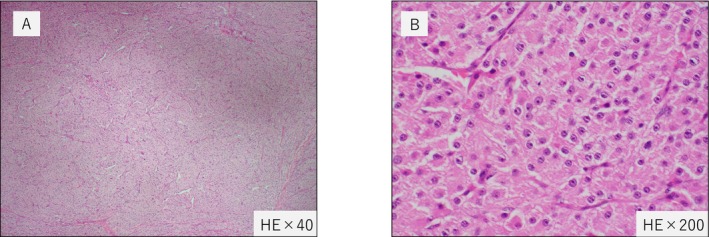
Pathological evaluation of LCT with hematoxylin and eosin (HE) staining. The diagnosis was Leydig cell tumor, characterized by a well‐defined, solid, trabecular growth pattern with eosinophilic granular cytoplasm and centrally located distinct round nuclei. Necrosis was observed within the tumor, with mitotic figures in up to 5/10 high‐power fields, with the presence of atypical mitotic figures. (A) ×40, (B) ×200.

The patient was a 43‐year‐old male with a chief complaint of right testicular induration and pain at the time of the initial examination. Testicular ultrasound revealed a hypoechoic area on the right side and increased blood flow. Contrast‐enhanced CT and bone scintigraphy revealed no metastases. Right orchiectomy was performed after 2 weeks. Pathologically, the tumor was diagnosed as a Leydig cell tumor (LCT). It also showed invasion into the testicular network, tumor necrosis, 5/10 HPF nuclear fission, and atypical nuclear fission (Figure 1). Blood tests showed no elevation of tumor markers in the testicular tumor after the operation (HCG‐β < 1.2 mIU/mL, AFP 2.8 ng/mL, LDH 175 U/L). After the initial diagnosis, CT performed 1 year and 7 months later revealed para‐aortic lymph node swelling. One month later, retroperitoneal lymph node dissection was performed, and the dissected lymph nodes were also consistent with the Leydig cell tumor. Postoperative follow‐up CT scans performed at 1 year and 10 months revealed additional mediastinal lymph node swelling. The decision to resect this new lesion was made after a review by both an internal multidisciplinary Cancer Conference and an external cancer board. The consensus was that complete surgical resection should be the primary treatment strategy and that early surgical intervention would be desirable given the unpredictable nature of further metastasis. Thoracoscopic lymph node excision was performed 2 years after the initial diagnosis.

**FIGURE 2:**
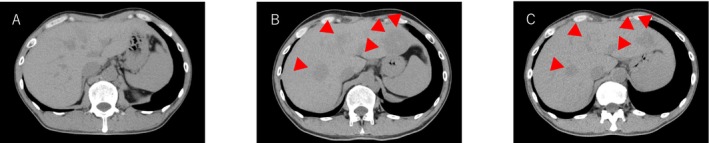
CT images of the present case: (A) Before the start of BEP treatment, (B) At the time of BEP discontinuation, showing evident liver metastasis, (C) 3 months after the initiation of mitotane, demonstrating a significant reduction in liver metastasis.

A CT scan performed 2 years and 2 months after the initial diagnosis revealed multiple liver metastases (Figure 2A). A cancer panel test (FoundationOne) was conducted at another institution; however, it did not identify any actionable drug targets for treatment. He was started on anticancer chemotherapy with bleomycin, etoposide, and cisplatin (BEP) based on criteria for ovarian sex cord‐stromal tumors. However, after four cycles, BEP was discontinued as a surveillance CT scan revealed progression of liver metastasis (Figure 2B); new enlarged lymph nodes and bone lesions were also detected. At this point, there was no elevation in HCG‐β or sex hormones (estradiol, progesterone, testosterone). Almost 3 years after the initial diagnosis, the patient was started on 1.5 g/day of mitotane. At week 1, his cortisol level was 10.8 μg/dL. The dose of mitotane was increased to 4.5 g/day at week 3. CT showed partial response of the metastases (63.5% reduction in liver metastasis, Figure 2C) 3 months after starting mitotane. Mitotane was reduced to 3 g/day at month 4 because of fatigue, anorexia, and nipple discomfort; however, persistent gastrointestinal toxicity required discontinuation of mitotane at month 5. Although tumor control was maintained, his serum cortisol dropped from 3.9 to 1.9 μg/dL; thus, prednisolone replacement was initiated at month 6. Simultaneously, mitotane was resumed at 3 g/day. However, the abdominal lymph nodes were enlarged, and further treatment was difficult by the 8th month. The patient died 4 years after the initial diagnosis and 14 months after the initiation of mitotane treatment.

## 3 Discussion

Sex cord‐stromal tumors are non‐seminomatous testicular tumors. They account for 4% of all testicular tumors, with Leydig cell tumors(LCTs) being the most common [1]. All pediatric cases of LCT and 90% of adult cases are noted to be benign [2]. They are characterized by painless testicular enlargement and may secrete steroid hormones such as testosterone [3]. In a small number of cases, loss of libido, erectile dysfunction, infertility, and testicular atrophy have been reported [3, 4]. Frankhauser et al. reviewed 1375 cases collected from published articles, and metastases were seen in 101 cases, most commonly in the retroperitoneal lymph nodes (60%), lungs (38%), and liver (29%) [5]. In multivariable regression analyses, older age (> 42 years), tumor size (> 30 mm), necrosis, and vascular‐lymphatic invasion were identified as risk factors for metastasis [5]. In a study of pathological findings in 19 LCTs (5 malignant cases) by Kim et al., the following features were correlated with malignant potential: diameter of ≥ 5 cm, infiltrative margins, lymphatic and vascular invasion, necrosis, mitoses > 3/10 HPF, and nuclear atypia (G2 or 3) [2]. In our case, while the tumor did not meet the criteria for size (< 5 cm), infiltrative margins, or vascular invasion, it exhibited both tumor necrosis and a high mitotic rate (5/10 HPF).

There are reports that both chemotherapy and radiotherapy have poor efficacy against metastatic tumors, and that retroperitoneal lymph node dissection (RPLND) is recommended for Stage I tumors in patients older than 35–40 years of age and in those with poor prognostic pathology [5, 6].

Treatment for malignant LCT includes radiotherapy, cisplatin‐based chemotherapy, mitotane, and lonidamine, but these treatments are associated with poor outcomes. A search of PubMed revealed 12 cases on the use of mitotane for metastatic LCTs [7, 8, 9, 10, 11, 12, 13, 14, 15, 16]. While the longest treatment duration with mitotane was 33 months, in most cases, it was discontinued after 2–3 months (Table 1). In our case, the total treatment duration was 7 months, with a notable reduction in tumor size.

**TABLE 1:** Overview of published mitotane case reports in LCT.

Ref.	Author, year	Case	Age (initial visit)	Metastatic sites (per original report)	Line of systemic therapy	Mitotane dose	Adverse events	Duration of mitotane treatment	Overall survival (measured from date of the initial diagnosis)
7	Chortis V, 2018	1	51	Liver, lungs, retroperitoneal lymph nodes	1st‐line	3 g/day	Not reported	10 months	About 190 months
7	Chortis V, 2018	2	59	Liver, kidney, peritoneum	1st‐line	2–4.5 g/day	Not reported	6 months	About 60 months
8	Azer PC, 1981	1	63	Lungs, retroperitoneal lymph nodes	1st‐line	2.4–14 g/day	Malaise, weakness, weight loss, nausea, vomiting	8 months	13 months
9	Feldman PS, 1982	1	51	Retroperitoneal mass, liver and right kidney	1st‐line	10 g/day	Not reported	About 11 months?	37 months
10	Davis S, 1981	1	61	Mediastinal & hilar lymph nodes, retroperitoneum	3rd‐line	12 g/day	Not reported	8 weeks	90 months
10	Davis S, 1981	2	74	Abdominal mass	3rd‐line	10 g/day	Not reported	Not reported	36 months
11	Schwarzman MI, 1989	1	52	Retroperitoneal mass, peritoneum, omemtum and small bowel	1st‐line	9 g/day	Anorexia, nausea	10 weeks	About 90 months
12	Grem JL, 1986	3	28	Liver	2nd‐line	1.5–4 g/day	Not reported	7 weeks	≥ 128 months
13	Bertram KA, 1991	1	60	Retroperitoneum, liver, bone (ribs, spine, lumbar & sacral vertebrae), mediastinal and para‐aortic lymph nodes and mesentery	2nd‐line	6–12 g/day	Nausea	< 2 months	About 104 months
14	Tamoney HJ, 1969	1	64	Para‐aortic & supraclavicular lymph nodes, lungs, liver	3rd‐line	10 g/day	CNS depression, coma	< 1 month	About 4 years
15	van der Hem KG, 1992	1	56	Retroperitoneal lymph nodes, liver	1st‐line	3–10 g/day	Chylous ascites, fatigue, poor appetite, diarrhea	About 33 months (intermittent)	81 months
16	Abelson D, 1966	1	55	Liver, abdominal wall, omentum, sternum	1st‐line	4 g/day	Epigastric distress, diarrhea, adrenal insufficiency	About 14 months	23 months

Mitotane has been widely adopted for the treatment of adrenocortical carcinoma (ACC) [7]. During human embryonic development, the gonads and adrenal glands are derived from the urogenital ridge, which led us to hypothesize that mitotane may also be effective against LCT.

During mitotane treatment, most patients require high‐dose oral steroid replacement therapy to prevent adverse events (e.g., skin, gastrointestinal, and central nervous system symptoms), along with adrenal hormone deficiency and gynecomastia [17, 18]. Regular monitoring of mitotane levels is recommended, as > 78% of patients taking doses of ≥ 2 g experienced side effects [19]. Although individual differences exist, patients typically reach the ideal plasma concentration (14–20 mg/L) after approximately 3 months of mitotane treatment (2–10 g/day) [18].

Although cancer‐related outcomes were not significantly affected, insufficient management of adverse events may have negatively influenced the patient's quality of life. In cases of long‐term administration, careful adjustment of the mitotane dosage is required to maintain an effective drug concentration.

## 4 Conclusion

In conclusion, the present case of malignant LCT treated with mitotane highlights the complexities in managing this rare and aggressive testicular cancer, contributing to ongoing discussions on LCT management and underscoring the importance of adaptable approaches to balance treatment effectiveness with adverse events. Further research is needed to improve the therapeutic strategies for LCT.

## Ethics Statement

This study was approved by the Institutional Review Board of Hyogo Prefectural Amagasaki General Medical Center (approval number 3‐38, on June 18, 2021).

## Consent

Written informed consent was obtained from all participants in this study.

## Conflicts of Interest

The authors declare no conflicts of interest.
